# Characterization of Enhancing MS Lesions by Dynamic Texture Parameter Analysis of Dynamic Susceptibility Perfusion Imaging

**DOI:** 10.1155/2016/9578139

**Published:** 2016-01-13

**Authors:** Rajeev K. Verma, Johannes Slotboom, Cäcilia Locher, Mirjam R. Heldner, Christian Weisstanner, Eugenio Abela, Frauke Kellner-Weldon, Martin Zbinden, Christian P. Kamm, Roland Wiest

**Affiliations:** ^1^Support Center for Advanced Neuroimaging, University Institute of Diagnostic and Interventional Neuroradiology, Inselspital, University of Bern, 3010 Bern, Switzerland; ^2^Tiefenau Hospital, Institute of Radiology, Spital-Netz Bern, 3004 Bern, Switzerland; ^3^Department of Neurology, Inselspital, University of Bern, 3010 Bern, Switzerland

## Abstract

*Purpose*. The purpose of this study was to investigate statistical differences with MR perfusion imaging features that reflect the dynamics of Gadolinium-uptake in MS lesions using dynamic texture parameter analysis (DTPA).* Methods*. We investigated 51 MS lesions (25 enhancing, 26 nonenhancing lesions) of 12 patients. Enhancing lesions (*n* = 25) were prestratified into enhancing lesions with increased permeability (EL+; *n* = 11) and enhancing lesions with subtle permeability (EL−; *n* = 14). Histogram-based feature maps were computed from the raw DSC-image time series and the corresponding texture parameters were analyzed during the inflow, outflow, and reperfusion time intervals.* Results*. Significant differences (*p* < 0.05) were found between EL+ and EL− and between EL+ and nonenhancing inactive lesions (NEL). Main effects between EL+ versus EL− and EL+ versus NEL were observed during reperfusion (mainly in mean and standard deviation (SD): EL+ versus EL− and EL+ versus NEL), while EL− and NEL differed only in their SD during outflow.* Conclusion*. DTPA allows grading enhancing MS lesions according to their perfusion characteristics. Texture parameters of EL− were similar to NEL, while EL+ differed significantly from EL− and NEL. Dynamic texture analysis may thus be further investigated as noninvasive endogenous marker of lesion formation and restoration.

## 1. Introduction

MR-based imaging biomarkers are integral parts of the diagnosis workup of multiple sclerosis since more than 20 years [1]. These biomarkers include baseline MRI lesion count, lesion load, and topography, as well as T1-associated signatures of axonal damage [2]. The most common phenotype of MS—relapsing-remitting MS—is characterized by recurrent perivenous inflammation and demyelination of brain tissue resulting in progressive neurological dysfunction triggered by immunopathogenic mechanisms that are not fully explored until now [3]. In particular, dysregulation and disruption of the blood-brain barrier (BBB) are a critical event in the pathological evolution of MS lesions [4]. Absence of Gd-enhancement does not preclude BBB breakdown and vice versa [5], although a temporal change of enhancement is frequently considered as a surrogate marker for BBB restoration. Thus, in daily clinical practice, most commonly the tissue is thus simply characterized as “enhancing” or “nonenhancing” and the dynamic aspects of lesion enhancement are frequently waived [6]. Beyond T1-weighted static MRI, perfusion imaging offers an alternative to quantify the amount of vascular permeability [7] and to analyze the time-dependency of the BBB disruption [8, 9]. Since perfusion imaging can be standardized according to the amount, flow, and timing of Gd-administration, lesion morphology may be reevaluated according to changes in microstructural perfusion and leakage during the first pass of the bolus passage. A recently proposed method, dynamic texture parameter analysis (DTPA), allows investigating these spatiotemporal characteristics to describe specific features of enhancing and nonenhancing lesions in MS [10]. DTPA enables a quantitative grading of MS lesions and discriminates lesions according to their statistical metrics. In this study, we aimed to investigate whether contrast agent extravasation is associated with characteristic metrics derived from dynamic textures of histograms during the first pass of the perfusion and early reperfusion. We hypothesized (i) that microstructural perfusion analysis can be used to subcategorize enhancing lesions according to their vascular permeability [11] and (ii) that statistical texture analysis segregates enhancing MS lesions by lesion-specific time-dependent patterns.

## 2. Materials and Methods

### 2.1. Patients

12 patients (9 women, 3 men) with relapsing-remitting MS (RR-MS, *n* = 9) and secondary progressive MS (SP-MS, *n* = 3) according to the revised McDonald criteria of 2010 [12] were included into this retrospective analysis. The 3 SPMS patients presented with an initial course of RRMS followed by stepwise deterioration with superimposed relapses. Median age was 43 y (range 23–74 years). All data were derived from an ongoing prospective study that incorporates perfusion MRI as part of the MS imaging protocol. Inclusion criteria were (i) at least one lesion with enhancement on T1-weighted images and (ii) at least one lesion without enhancement on T2/FLAIR images and normal hematocrit (0.34–0.47) [13]. The study was approved by the local ethics committee (Cantonal Ethics Commission Bern, Switzerland). All patients gave written informed consent to participate in this study.

### 2.2. MRI Sequences and Parameters

All subjects underwent an MRI examination with the same 3 T MRI system (Siemens Magnetom Trio, Siemens AG, Erlangen, Germany) equipped with a 32-channel head coil. The entire MS protocol encompassed (i) diffusion weighted imaging (TR 6100 ms, TE 102 ms, FoV read 230 mm, FoV phase 100%, voxel size 1.8 × 1.8 × 4.0 mm, acquisition time 1 : 45 min. 19 parallel images with a slice thickness of 4.0 mm), (ii) T1-weighted MPR pre- and postgadobutrol i.v. (TR 2530 ms, TE 2.96 s, FoV read 250 mm, FoV phase 87.5%, voxel size 1.0 × 1.0 × 1.0 mm, flip angle 7°, acquisition time 4 : 30 min, slices per slab 160, and slice thickness 1.0 mm), (iii) T2-weighted imaging (TR 6580 ms, TE 85 ms, FoV read 220 mm, FoV phase 87.5%, voxel size 0.7 × 0.4 × 3.0 mm, flip angle 150°, and acquisition time 6 : 03 min. 42 parallel images were acquired with a slice thickness of 3.0 mm), (iv) 3D FLAIR imaging (TR 5000 ms, TE 395 ms, FoV read 250 mm, FoV phase 100%, voxel size 1.0 × 1.0 × 1.0 mm, and acquisition time 6 : 27 min. 176 parallel images were acquired with a slice thickness of 1.0 mm), and (v) T1-weighted imaging postgadobutrol i.v. (TR 297 ms, TE 2.67 ms, FoV read 220 mm, FoV phase 87.5%, voxel size 0.8 × 0.6 × 3.0 mm, flip angle 70°, and acquisition time 4 : 14 min. Forty-two parallel images were acquired with a slice thickness of 3.0 mm). All patients received gadobutrol (Gadovist) 0.1 mL·kg^−1^ bodyweight. The flow rate was 5 mL/s, followed by 20 mL of sodium chloride with the same flow rate. Patients were positioned comfortably in the head coil and padding on either side of the head was used to help immobilization. The intravenous line with a long tube was put before examination to avoid unnecessary MRI table moving during data acquisition. Perfusion analysis was performed using DSC in addition to the standard sequences in all patients (TR 1400 ms, TE 29 ms, averages 1, FoV read 230 mm, FoV phase 100%, voxel size 1.8 × 1.8 × 5.0 mm, flip angle 90°, 80 repetitions, and acquisition time 1 : 59 min. 19 parallel images were acquired with a slice thickness of 5.0 mm).

### 2.3. Preclassification of Enhancing and Nonenhancing Lesions for Texture Analysis

Demyelinating lesions were identified on T2-weighted and fluid attenuated inversion recovery (FLAIR) MR-images. Further enhancing supratentorial lesions were identified in the T1-weighted sequence after Gd administration. To compare active lesions with inactive lesions in the perfusion images, at least one supratentorial nonenhancing lesion per patient was selected for comparison within the same vascular territory.

### 2.4. Preclassification of Gd-Enhancing Lesions according to Their Permeability

**Figure 1:**
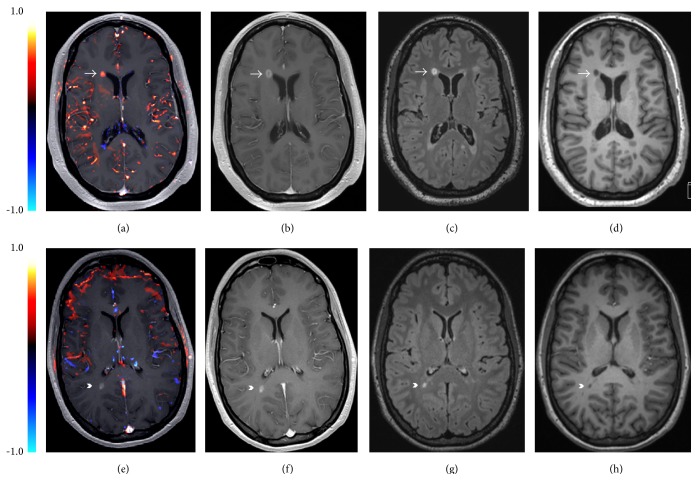
Prestratification of demyelinating lesions according to their leakiness. The upper row shows an enhancing lesion classified as EL+ (arrow), (a) T1w post-Gd after postprocessing with NORDIC Ice: the red area reflects the lesion with high permeability above the predefined cutoff value of 0.01; (b) T1w post-Gd; (c) FLAIR sequence; (d) T1w pre-Gd. The lower row shows an enhancing lesion classified as EL− (arrowhead); (e) T1w post-Gd, after postprocessing with NORDIC Ice: the lesion was classified as low permeability lesion below the predefined cutoff value of 0.01; (f) T1w post-Gd; (g) FLAIR sequence; (h) T1w pre-Gd.

To determine the effect of leakage on postcontrast T1-weighted MPR images, we used a commercially available software (NordicIce Version 2.3; NordicNeuroLab AS, Bergen, Norway). We preselected enhancing MS lesions according to their leakage coefficient K2 following Boxerman et al. [11], a correction method in which contrast extravasation is estimated in each voxel by determining the voxel-wise deviation from a “nonleaky” reference tissue response curve. K2 refers to the leakage rate detected during DSC imaging. The method utilizes linear fitting to determine the leakage coefficient, a first-order estimate of vascular permeability proportional to the leakage, the product of permeability, and the surface area. In short, this method assumes that the contrast agent exhibits T2 or T2^*∗*^ effects (“negative contrast effect”) in the intravascular compartment but assumes that the contrast effect is mainly driven by T1-shortening once the agent leaks into the extracellular space (“positive contrast effect”). The K2 measured with DSC perfusion MR imaging reflects a combination of all these factors on vascular leakiness. The K2 estimation has been previously employed to investigate differences in vascular permeability between gliomas of different grades and between primary CNS lymphomas and glioblastoma multiforme [7, 11]. For further texture analysis within the perfusion images, lesions were subdivided into enhancing lesions with a detectable K2 cutoff that exceeded 0.010, indicating increased permeability and enhancing lesions with a K2 cutoff lower than 0.010, indicating low permeability resembling normal appearing white matter, as suggested in a previous study of patients with cerebral gliomas [7] (see Figure 1).

### 2.5. Dynamic Texture Parameter Analysis

The concept of a texture refers to the appearance of a tissue defined by its shape, composition, arrangement, and proportion of its elementary parts. DTPA focuses on a quantification of regional tissue inhomogeneity according to its individual texture during the bolus passage of Gd. The method uses a model-free approach to analyzing MR texture parameter maps at different time points between the first recorded image during the baseline and the subsequent images during bolus passage. The bolus passage was further divided into three epochs, namely, the inflow, the outflow, and the reperfusion time periods following a previous study to investigate lesion effects and leakage on the capillary level separately for arteries and veins [14]. The inflow and outflow time intervals are patient-dependent; they depend on the patient cardiac health state but also on the vascular state of the patient (e.g., stenosis). The inflow period was in the order 2 to 3 seconds; the outflow period was a little longer around 3–5 seconds. The baseline period was defined as the period between the start of the bolus injection and the time point where 2 subsequent data points exceeded 3 standard deviations of the concentration curve noise level. The inflow period was defined as end of the baseline period to the peak of the concentration time curve. The outflow period was defined as 1st time point after the peak maximum to the first local minimum. The recirculation period encompasses the 1st time point after the local minimum until the last image. To facilitate interindividual comparisons and to account for noise and image nonuniformity due to magnetic field inhomogeneity, a twofold normalization procedure has been performed. The normalization consisted of (i) a normalization of the normal appearing white matter (NAWM) in the frontal white matter reference region to the numerical value of 1000, followed by (ii) a normalization of the time integral of NAWM over encompassing the inflow and outflow period which was set to a reference value of 200. A detailed mathematical description of the computational procedure is provided in [10].

Texture parameter maps (TPMs) were computed from the* raw* DSCE-images using an in-house developed computer JAVA-application. The original raw DSC EPI image series constitutes a texture parameter map itself and was further denoted by “TPM-ORIG.” The* difference* image time series computed from TPM-ORIG were denoted by “TPM-DIFF” and calculated by a subtraction of the first steady state baseline image from every subsequent image during bolus passage. Additionally, we calculated the TPM-standard deviation “TPM-SD” and TPM-variance “TPM-VAR” maps. The TPM-SD and TPM-VAR maps were computed from the TPM-DIFF map by computing pixel-by-pixel the pixels* local* standard deviation and* local* variance for a 5 × 5 pixel region. These maps are thus computed in the same fashion as one would compute a moving average filtered version of an image. The regions of interest (ROI) were manually segregated by a board certified neuroradiologist on the raw images and copied to the TPMs. For each TPM we calculated the following* statistical* parameters, that is, the mean intensity (“mean”), standard deviation (“SD”), variance (“VAR”), and variance of variance (“VARVAR”). For instance, the mean value of a region defined in the TPM-SD measures the average local standard deviation of the TPM-DIFF-map and hence may act as a surrogate marker for tissue heterogeneity. The other statistical parameters (SD, VAR, etc.) are features that measure higher order statistical properties of the TPMs.

### 2.6. Statistical Analysis

We used the statistical software SPSS (IBM Corp. Released 2011. IBM SPSS Statistics for Windows, Version 20.0. Armonk, NY: IBM Corp., USA) for the statistical analysis of the acquired data. We aimed to investigate which TPMs differentiate lesions according to severe versus marginal leakage.

Differences between nonenhancing inactive lesions (NEL), enhancing lesions with increased permeability (EL+), and enhancing lesions with subtle permeability (EL−) as determined by their K2 cutoff were analyzed. First, we analyzed the statistical distribution of all TPM in order to be able to select the correct test statistics. A WELCH-ANOVA was performed for all TPM within the prestratified epochs (baseline, inflow, outflow, and reperfusion period) due to heteroscedasticity. For post hoc multiple comparisons between NEL, EL+, and EL−, the Games-Howell method was selected for all texture parameters at a given *p* value of *p* < 0.05 in the WELCH-ANOVA.

## 3. Results

The Expanded Disability Status Scale (EDSS) of the 12 patients (9 female; median age 43 y) ranged between 1 and 7.5 (mean 3.83, SD 1.95). A detailed description of the clinical data is provided in Table 1. Nine of 12 patients were drug naive, the remaining 3 were treated with stable dosage of disease-modifying drugs (interferon 1b or glatiramer acetate). Eight of 12 patients showed acute neurological symptoms, while the remaining 4 showed none. The active lesions were located in the deep white matter (3), juxtacortical (6), and periventricular (16). The NEL were selected pairwise from the corresponding regions of the EL.

A total of 52 lesions were identified (26 EL and NEL). One EL had to be withdrawn from final analysis due to an equivocal Gd-uptake, resulting in 51 lesions available for final analysis. The 25 enhancing lesions were subdivided into 11 EL+ and 14 EL− according to a K2 cutoff value of 0.01. The average lesion size in this study was 146.62 mm^3^ (±95.82) for NEL, 156.59 mm^3^ (±154.29) for EL+, and 143.00 mm^3^ (72.65) for EL−, with no significant volume differences among the three cohorts. The average lesion size was 9 voxels (1.8 × 1.8 × 5 mm). A multivariate analysis was performed on the features extracted from the four texture parameter maps (TPM-ORIG, TPM-DIFF, TPM-VAR, and TPM-SD): for this analysis the within-lesion mean intensity (mean), standard deviation (SD), variance (VAR),* skewness*, and* kurtosis* values were analyzed. A one-way ANOVA with Welch correction identified 19/48 TPM features that discriminated among the 3 lesion subtypes (Table 2).

The TPMs that appeared most sensitive to discriminate EL+ and EL− were TPM-DIFF (7 features), followed by TPM-SD (5 features) and TPM-VAR (5 features). The major effects were observed during late perfusion epochs, outflow (5), and reperfusion (13). A detailed description is provided in Table 4.

A post hoc Games-Howell test indicated significant differences between EL+ and NEL in 8 and between EL+ and EL− for 6 features (Table 3). The strongest discriminators between EL+ versus NEL and EL− were observed during reperfusion (9 features) and outflow (5 features). EL− and NEL were discriminated exclusively by the TPM-SD during outflow. No single test discriminated between all the three subgroups. The results are summarized in Table 3.

## 4. Discussion

**Figure 2:**
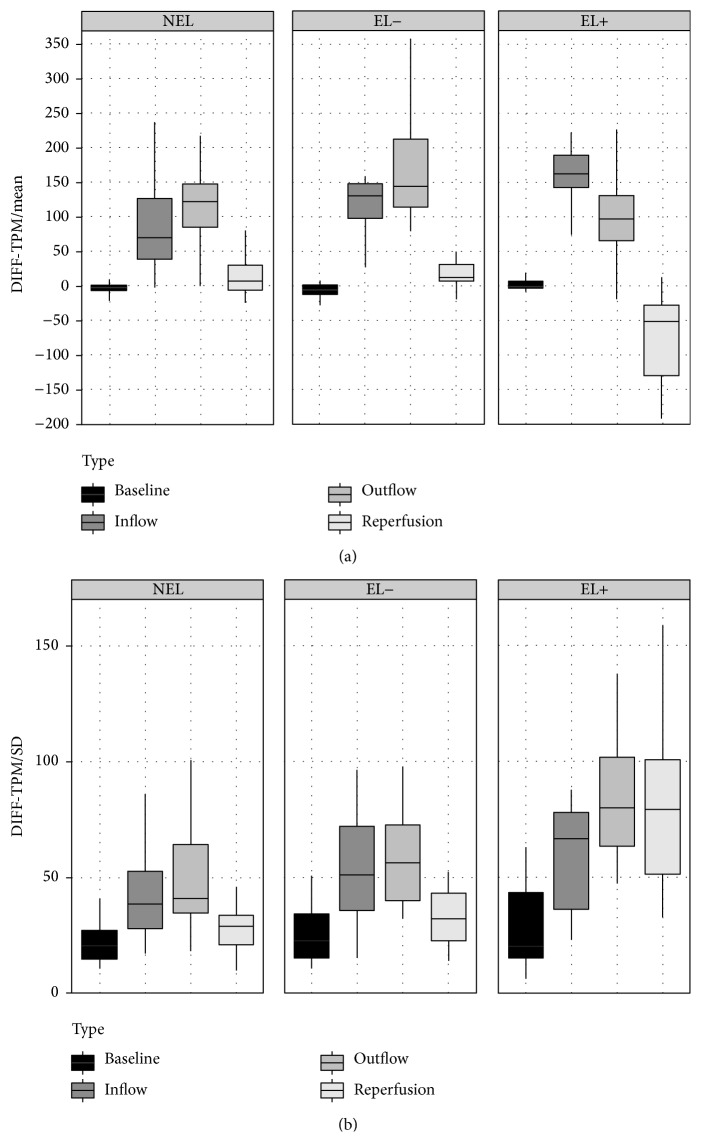
The averaged temporal dynamics of NEL, EL−, and EL+ are exemplarily provided for the mean (a) and SD (b) of the DIFF-TPM. (a) Mean of the DIFF-TPM for NEL, EL−, and EL+: the bars reflect the perfusion intensity of the lesion subtypes during baseline, inflow, outflow, and reperfusion. Significant differences between EL+ and NEL are detected during the inflow (*p* = 0.009) and between EL+ and EL− (*p* = 0.018) and EL+ and NEL (*p* = 0.019) during the reperfusion. The mean values for NEL and EL− increase until the end of the OF with subsequent normalization to baseline during the RP. In contrast the mean values of EL+ increase only until IF, followed by a decrease during outflow and reperfusion with negative values during reperfusion due to local leakage effects. (b)* SD* of the DIFF-TPM for NEL, EL−, and EL+: the bars reflect the perfusion homogeneity of the lesion subtypes during baseline, inflow, outflow, and reperfusion. The temporal dynamics of the EL− are similar to that of the NEL, indicating similar perfusion characteristics of EL− and NEL (n.s.). EL+ are characterized by increasing inhomogeneity during outflow and reperfusion. The SD segregated EL+ from EL− during OF (*p* = 0.029) and RP (*p* = 0.044) and EL+ from NEL during OF (*p* = 0.003) and RP (*p* = 0.03). The persistence of increased SD during RP indicates local leakage effects as observed in (a).

DTPA enables a* quantitative* tissue characterization of MS lesions based on histogram-based textural features. Previous studies investigated the feasibility of contrast-free static and contrast-enhanced dynamic perfusion texture analyses to differentiate EL from NEL [10, 15]. Here, we demonstrated that EL can be further categorized into EL+ and EL− and that EL− behave similarly to NEL by post hoc analysis of texture parameters derived from DSC perfusion imaging. The dynamic texture features of EL+ and EL− correlated with the amount of vascular permeability, reflecting predominantly statistic differences in the local texture dynamics during outflow and reperfusion. The texture parameter changes are statistical measures that segregated lesions visually overall classified as “enhancing MS plaques.” The mean contrast differences and standard deviations of the computed texture parameter maps were remarkably different between EL+ and EL− and the derived features reflect the net effect of the contrast extravasation on the dynamic signal intensity curves. In contrast, kurtosis and skewness did not differ between the two cohorts, indicating that only first- and second-order moments had discriminative power and that steepness and asymmetry of the contrast agent distribution played a less important role in our analysis. Beyond a statistically significant T2^*∗*^ effect caused by intralesional extravasation of Gd during outflow and reperfusion, significantly increased Gd concentrations and accelerated inflow were observed in EL+ compared to NEL. Both may reflect a net inflammation-related vasodilatation in acute and more aggressive lesions. There was a strong similarity in the textures of EL− and nonenhancing inactive lesions, reflecting a delayed Gd peak concentration during venous outflow (Figure 2(a)), with only subtle differences in the TPM-DIFF for “mean” and “SD.”

Pathological features that encompass the evolution of acute versus subacute Gd-enhancing MS lesions have been recently investigated by high-resolution dynamic contrast-enhanced MRI [9]. Longitudinal enhancement dynamics of initially nodular lesions revealed a centrifugal pattern while older ring-like lesions enhanced centripetally with delayed lesion filling. The findings indicate lesions grow outward from a disrupted BBB along the central vein with a secondary opening of the BBB in peripheral vessels. Later, partial closure of BBB along the central vein and its contiguous vessels results in a reduction of the central enhancement and/or reduction in perfusion of the lesion core. The DTPA features may reflect similar changes in lesion formation from an early disruptive process continuously into the late stage of a hypometabolic plaque. The tissue response to plaque formation encompasses an inflammatory response and may end up in an impaired microcirculation in late stages of EL− and after closure of the BBB in nonenhancing inactive lesions. Dynamic enhancement data may thus offer a time-effective alternative for a more detailed characterization of the stages of lesion development.

This study has limitations: We have currently not investigated longitudinal DTPA characteristics to follow whether characteristics of EL+ turn into EL− and NEL over time, which will be substance of subsequent investigations. We selected a limited number of patients with RR-MS and relapsing SP-MS that were stratified into EL+ and EL− based on a preselection of T1 Gd-enhancing lesions according to their vascular permeability. This enabled us to identify texture features of lesions with high versus low- or nonpermeable lesions. DTPA does not require a perfusion model such as deconvolution methods or model-fitting of the bolus passage function for quantification of the DSC image series. However, the required normalization procedure may be affected by local T1 effects due to increased permeability in the periventricular NAWM. In order to minimize this effect, we normalized the data by setting the reference region for the normalization into the NAWM close to the gray/white matter border zone with a maximum spatial distance to the MS lesions.

DTPA features reflect statistic properties of enhancing MS lesions beyond descriptions of “*enhancement*” or “*no enhancement*” as currently used in daily routine. The technique identifies characteristic textural features that appear during lesion evolution from severe inflammation to recovery. Noteworthy, even though EL− are classified as active lesions in daily practice, their perfusion characteristics in terms of dynamic texture changes resemble that of NEL. This is a novel finding in MS that motivates the incorporation of these features into machine learning approaches, for example, into decision forest classifiers that can handle high-dimensional input data in larger datasets. Our data further support previous findings of tissue dependency in microcirculation [10] that may be further extended into the refinement and differentiation of white matter lesions other than MS in future.

## Figures and Tables

**Table 1:** Detailed patient information.

Pt. number	Sex	Diagnosis	Age (years)	EDSS	Disease duration	Therapy	Acute disease exacerbation/start before MRI	Symptoms of acute disease exacerbation
1	F	RR-MS	56	3	29 y	No	No	—
2	F	RR-MS	44	4	First relapse 5 months ago	No	No	—
3	F	RR-MS	23	1	4.5 months	No	No	—
4	F	SP-MS	35	5	14 y	No	Yes/5 months	Mild paresis left leg/impaired walking
5	F	RR-MS	42	4	5 months	No	Yes/2–5 months	Tetraspasticity/urinary urgency
6	F	SP-MS	60	7.5	19 y	No	Yes/2 d	Subacute hemiparesis left
7	F	RR-MS	50	3.5	8 y	Yes (interferon beta 1b)	No	—
8	M	SP-MS	74	7	25 y	No	Yes/1 d	Worsening of paraparesis
9	F	RR-MS	44	4	22 months	No	Yes/3 weeks	Vertigo, weakness in right leg, tongue sensation left
10	M	RR-MS	30	1.5	12 months	Yes (interferon beta 1b)	Yes/10 d	Weakness in left leg and arm
11	M	RR-MS	28	2.5	7 months	No	Yes/3 months	Retrobulbar pain, eye lid twinkles
12	F	RR-MS	24	3	3.5 y	Yes (glatiramer acetate)	Yes/1 month	Urinary urgency/vertigo

Note: RRMS: relapsing-remitting MS, SPMS: secondary progressive MS, and EDSS: Expanded Disability Status Scale.

**Table 2:** One-way ANOVA of the texture parameter maps (TPMs): number of significant differences in the different time periods. A total of 19 out of 48 (12 × 4) tests revealed statistical significant differences.

	IF	OF	RP	Total
TPM-ORIG	0	0	2	2
TPM-DIFF	1	2	4	7
TPM-SD	0	1	4	5
TPM-VAR	0	2	3	5
Total	1	5	13	19

Note: ORIG: raw image, DIFF: difference image, SD: standard deviation, VAR: local variance, IF: inflow, OF: outflow, and RP: reperfusion.

**Table 3:** Post hoc analysis (Games-Howell test) of all 19 texture parameter maps (TPMs) that discriminated significantly between EL+, EL−, and NEL in one-way ANOVA.

	Stat. par.	Time period	*p* values			
			ANOVA	EL+ versus EL−	EL+ versus NEL	EL− versus NEL
TPM-ORIG	SD	RP	<0.001^*∗*^	0.018^**∗**^	0.035^**∗**^	0.695
TPM-ORIG	VAR	RP	<0.001^**∗**^	0.035^**∗**^	0.05	0.82
TPM-DIFF	Mean	IF	0.02^**∗**^	0.117	0.009^**∗**^	0.356
TPM-DIFF	Mean	RP	<0.001^**∗**^	0.018^**∗**^	0.019^**∗**^	1
TPM-DIFF	SD	OF	<0.001^**∗**^	0.029^**∗**^	0.003^**∗**^	0.429
TPM-DIFF	SD	RP	<0.001^**∗**^	0.044^**∗**^	0.030^**∗**^	0.323
TPM-DIFF	VAR	OF	0.03^**∗**^	0.084	0.030^**∗**^	0.642
TPM-DIFF	VAR	RP	0.006^**∗**^	0.248	0.231	0.254
TPM-DIFF	VARVAR	RP	0.039^**∗**^	0.295	0.332	0.967
TPM-SD	Mean	OF	<0.001^**∗**^	0.2	0.013^**∗**^	0.022^**∗**^
TPM-SD	Mean	RP	<0.001^**∗**^	0.032^**∗**^	0.012^**∗**^	0.063
TPM-SD	SD	RP	0.06^**∗**^	0.247	0.209	0.641
TPM-SD	VAR	RP	0.037^**∗**^	0.396	0.388	0.786
TPM-SD	VARVAR	RP	0.023^**∗**^	0.412	0.301	0.295
TPM-VAR	Mean	OF	0.006^**∗**^	0.364	0.128	0.119
TPM-VAR	Mean	RP	0.002^**∗**^	0.201	0.167	0.11
TPM-VAR	SD	OF	0.035^**∗**^	0.479	0.231	0.372
TPM-VAR	SD	RP	0.022^**∗**^	0.353	0.331	0.385
TPM-VAR	VARVAR	RP	0.03^**∗**^	0.373	0.368	0.517

Note: ORIG: raw image, DIFF: difference image, SD: standard deviation, VAR: local variance, IF: inflow, OF: outflow, RP: reperfusion, EL+: enhancing lesions with increased permeability, EL−: enhancing lesions with subtle permeability, and NEL: nonenhancing inactive lesions; ^*∗*^ *p* < 0.05.

**Table 4:** ANOVA of texture parameter map (TPM) between enhancing lesions with increased permeability (EL+), enhancing lesions with subtle permeability (EL−), and nonenhancing lesions (NEL) during the inflow, outflow, and reperfusion phase of Gadolinium.

	Inflow phase			Outflow phase			Reperfusion phase		
	EL+	EL−	NEL	EL+	EL−	NEL	EL+	EL−	NEL
TPM-ORIG mean	1304,21 (192,25)	1362,85 (255,7)	1355,87 (189,43)	1345,29 (168,13)	1323,95 (278,04)	1315,39 (184,87)	1471,50 (213.27)	1407,63 (249,18)	1371,52 (189,25)
TPM-ORIG SD	81,63 (35,88)	71,61 (25,22)	83,14 (23,29)	105,42 (47,76)	83,22 (24,63)	82,93 (24,08)	104,67^**∗∗∗**^ **(36,23)**	65,90^**∗∗∗**^ **(23,29)**	71,70^**∗∗∗**^ **(18,79)**
TPM-ORIG VAR	8398,22 (7762,47)	6118,53 (3594,62)	7692,92 (4430,34)	14007,4 (13771,01)	7848,0 (4205,91)	7680,92 (4266,07)	12839,71^**∗∗∗**^ **(8477,35)**	5122,31^**∗∗∗**^ **(3449,82)**	5762,78^**∗∗∗**^ **(2903,61)**
TPM-ORIG VARVAR	9,292*E* + 10 (2,135*E* + 11)	2,733*E* + 11 (5,176*E* + 11)	4,189*E* + 10 (1,042*E* + 11)	1,052*E* + 11 (2,371*E* + 11)	2,516*E* + 11 (4,952*E* + 11)	3,222*E* + 10 (7,731*E* + 10)	1,275*E* + 11 (2,783*E* + 11)	3,686*E* + 11 (7,462*E* + 11)	4,476*E* + 10 (1,103*E* + 11)

TPM-DIFF mean	169,36^**∗**^ **(52,72)**	125,37^**∗**^ **(53,97)**	94,75^**∗**^ **(85,2)**	96,31 (76,2)	169,7 (76,13)	134,37 (87,81)	−93,89^**∗∗∗**^ **(109,68)**	17,27^**∗∗∗**^ **(24,99)**	16,96^**∗∗∗**^ **(46,23)**
TPM-DIFF SD	63,13 (34,76)	53,11 (25,51)	44,18 (22,47)	83,95^**∗**^ **(26,98)**	57,61^**∗**^ **(20,25)**	48,63^**∗**^ **(24,36)**	97,74^**∗∗∗**^ **(76,73)**	32,44^**∗∗∗**^ **(12,4)**	26,98^**∗∗∗**^ **(9,42)**
TPM-DIFF VAR	5615,45 (5691,76)	4037,2 (3707,4)	2925,68 (3141,22)	8298,77^**∗∗**^ **(5176,78)**	4283,61^**∗∗**^ **(2875,78)**	3338,1^**∗∗**^ **(3687,26)**	16077,65^**∗∗**^ **(28558,66)**	1317,18^**∗∗**^ **(910,2)**	890,35^**∗∗**^ **(569,62)**
TPM-DIFF VARVAR	362978351 (781954883)	565232636 (1,2519*E* + 9)	81051320 (361085909)	158928026 (352922644)	579771510 (1,4200*E* + 9)	115586359 (491925502)	11124685^**∗**^ **(20663894)**	1204394^**∗**^ **(1989839)**	1585312^**∗**^ **(7228998)**

TPM-SD mean	82,02 (78,91)	61,28 (29,69)	46,59 (20,58)	95,3^**∗∗∗**^ **(41,8)**	70,25^**∗∗∗**^ **(21,55)**	49,64^**∗∗∗**^ **(23,93)**	90,54^**∗∗∗**^ **(57,66)**	37,84^**∗∗∗**^ **(13,24)**	28,11^**∗∗∗**^ **(11,25)**
TPM-SD SD	19,96 (20,99)	12,84 (6,88)	13,69 (12,19)	20,06 (11,41)	15,75 (7,15)	12,86 (6,74)	18,19^**∗∗**^ **(21,73)**	6,92^**∗∗**^ **(2,48)**	6,16^**∗∗**^ **(2,63)**
TPM-SD VAR	894,6 (2044,61)	278,93 (337,1)	383,08 (893,47)	608,14 (70,66)	363,77 (314,13)	244,75 (249,86)	832,13^**∗**^ **(1880,55)**	60,47^**∗**^ **(45,58)**	50,63^**∗**^ **(45,07)**
TPM-SD VARVAR	133699885 (432101281)	14178164 (44203559)	333236 (995759)	43079835 (126417365)	9755466 (21630255)	546108 (2004049)	2789669^**∗**^ **(5828131)**	432494^**∗**^ **(1010409)**	24796^**∗**^ **(82408)**

TPM-VAR mean	14218,05 (31070,18)	5783,47 (7305,58)	3360,02 (3458,39)	12234,88^**∗∗**^ **(13142,85)**	6401,44^**∗∗**^ **(4283,4)**	3598,19^**∗∗**^ **(4019,22)**	12809,09^**∗∗**^ **(19700,63)**	1755,86^**∗∗**^ **(1167,26)**	1022,15^**∗∗**^ **(871,07)**
TPM-VAR SD	6809,39 (15272,13)	2386,8 (2847,22)	2130,69 (3203,33)	5052,86^**∗**^ **(5789,93)**	2865,72^**∗**^ **(2165,8)**	1916,57^**∗**^ **(2061,01)**	5659,71^**∗**^ **(11524,87)**	611,76^**∗**^ **(395,44)**	441,39^**∗**^ **(377,58)**
TPM-VAR VAR	303820710 (973974129)	23181513,3 (62790956,2)	19406582,6 (57926284,0)	93586953,5 (243511997)	21479750,6 (40452799,7)	11376694,4 (23205702,7)	183622050 (557625048)	618965,14 (840273,06)	407992,02 (832069,7)
TPM-VAR VARVAR	4,156*E* + 18 (1,378*E* + 19)	6,431*E* + 16 (2,477*E* + 17)	4,414*E* + 14 (1,405*E* + 15)	7,405*E* + 17 (2,45*E* + 18)	2,636*E* + 16 (9,942*E* + 16)	1,017*E* + 15 (4,414*E* + 15)	1,625**E** + 15^**∗**^ (3,8**E** + 15)	1,165**E** + 13^**∗**^ (3,658**E** + 13)	1,018**E** + 12^**∗**^ (4,554**E** + 12)

Note: values are mean (SD); significant ANOVA results are marked with *∗* if *p* < 0.05 and with *∗∗* if *p* < 0.01 and with *∗∗∗* if *p* < 0.001.

## Abbreviations

BBB: Blood brain barrier
CNS: Central nervous system
DIFF: Difference image
DSC: Dynamic susceptibility contrast perfusion imaging
DTPA: Dynamic texture parameter analysis
EDSS: Expanded Disability Status Scale
EL+: Enhancing lesions with increased permeability
EL−: Enhancing lesions with subtle permeability
FoV: Field of view
Gd: Gadolinium
IF: Inflow phase
T1w MPRage: T1-weighted magnetization prepared rapid gradient echo sequence
MS: Multiple sclerosis
NAWM: Normal appearing white matter
NEL: Nonenhancing lesions
OF: Outflow phase
ORIG: Raw image
RP: Reperfusion phase
RRMS: Relapsing remitting multiple sclerosis
SD: Standard deviation
SPMS: Secondary progressive multiple sclerosis
TE: Echo time
TPM: Texture parameter maps
TR: Repetition time
VAR: Variance
VARVAR: Variance of variance.
